# Printed Electrochemical Strip for the Detection of miRNA-29a: A Possible Biomarker Related to Alzheimer’s Disease

**DOI:** 10.1021/acs.analchem.2c03542

**Published:** 2022-11-01

**Authors:** Antonella Miglione, Ada Raucci, Jussara Amato, Simona Marzano, Bruno Pagano, Tiziana Raia, Marco Lucarelli, Andrea Fuso, Stefano Cinti

**Affiliations:** † Department of Pharmacy, University of Naples “Federico II”, Via Domenico Montesano 49, 80131 Naples, Italy; ‡ Department of Experimental Medicine, Sapienza University of Rome, Viale Regina Elena 324, 00161 Rome, Italy; § Pasteur Institute Cenci Bolognetti Foundation, Sapienza University of Rome, Viale Regina Elena 291, 00161 Rome, Italy; ∥ BAT Center—Interuniversity Center for Studies on Bioinspired Agro-Environmental Technology, University of Naples “Federico II”, 80055 Naples, Italy

## Abstract

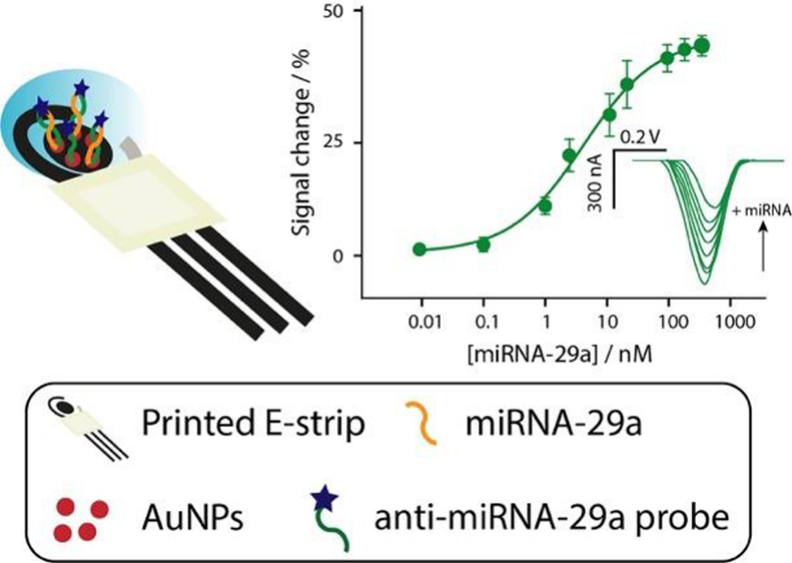

The development of electrochemical strips, as extremely powerful diagnostic tools, has received much attention in the field of sensor analysis and, in particular, the detection of nucleic acids in complex matrixes is a hot topic in the electroanalytical area, especially when directed toward the development of emerging technologies, for the purpose of facilitating personal healthcare. One of the major diseases for which early diagnosis is crucial is represented by Alzheimer’s disease (AD). AD is a progressive neurodegenerative disease, and it is the most common cause of dementia worldwide. In this context microRNAs (miRNAs), which are small noncoding RNAs, have recently been highlighted for their promising role as biomarkers for early diagnosis. In particular, miRNA-29 represents a class of miRNAs known to regulate pathogenesis of AD. In this work we developed an electrochemical printed strip for the detection of miRNA-29a at low levels. The architecture was characterized by the presence of gold nanoparticles (AuNPs) and an anti-miRNA-29a probe labeled with a redox mediator. The novel analytical tool has been characterized with microscale thermophoresis and electrochemical methods, and it has been optimized by selection of the most appropriate probe density to detect low target concentration. The present tool was capable to detect miRNA-29a both in standard solution and in serum, respectively, down to 0.15 and 0.2 nM. The platform highlighted good repeatability (calculated as the relative standard deviation) of ca. 10% and satisfactory selectivity in the presence of interfering species. This work has the objective to open a way for the study and possible early diagnosis of a physically and socially devastating disease such as Alzheimer’s. The results demonstrate the suitability of this approach in terms of ease of use, time of production, sensitivity, and applicability.

According to the World Health Organization (WHO), Alzheimer’s disease (AD) is the most common cause of dementia worldwide. It is a progressive neurodegenerative disease, characterized by neuronal death, loss of synaptic function, and atrophy in different brain areas, with loss of cognitive function and memory.^1−4^ Although no treatments that arrest AD are currently available, diagnosis at the predementia stage would represent a major breakthrough from therapeutic and prevention standpoints, with the possibility to reduce the risk of AD by 30%.^5^ In the extracellular space, amyloid β (Aβ) tends to deposit in insoluble fibrillar aggregates, which lead to the formation of the so-called amyloid plaques, playing a crucial role in initiating the disease and triggering a complex pathological cascade, which leads to a reactive inflammatory process that irreversibly damages neurons.^6,7^

Two forms of AD have been identified: familial AD, also known as early-onset AD (EOAD), and sporadic AD, characterized by late-onset AD (LOAD). About 70% of the risk of developing FAD may be due to mutations in the APP and PSEN1 genes.^8^ However, little is known about the mechanisms that lead to Aβ accumulation in the vast majority of sporadic AD cases.^9^

Although a paradigm shift from major hallmarks (e.g., amyloid β plaques, neurofibrillary tangles) to biomarker detection (e.g., Aβ42, p-tau, t-tau) in cerebrospinal fluid has been highlighted, an invasive lumbar puncture from the lower back is needed for sampling.^10^ Nowadays, AD diagnosis is usually performed through neurological investigations, by conducting cognitive tests^11^ and by imaging techniques,^12^ mainly magnetic resonance imaging (MRI), positron emission tomography (PET), and near-infrared (NIR), used to detect abnormalities in patient brains.^13^ The analysis of cerebrospinal fluid (CSF) and blood plasma biomarkers by immunohistochemistry and enzyme linked immunosorbent assay (ELISA) have also been used.^14^ These techniques have great limitations currently: they are time-consuming, expensive, and invasive and do not constitute a generalized method for early detection of AD. There is an urgent need for cheap and minimally invasive tests to more easily and accurately screen and identify patients at the earliest AD stages. In particular, access to the bloodstream opens up a revolutionary chance for the early diagnosis and surveillance of AD evolution (essential for personalized medicine) and timely/comprehensive monitoring of therapy efficacy. Among noninvasive AD biomarkers, microRNAs (miRNAs) may show a promising potential. They are found in circulatory biofluids, such as blood, and their diagnostic properties have been highlighted in several studies.^15^ They are also implicated in various neurodegenerative diseases such as AD, Huntington’s disease, Parkinson’s disease, amyotrophic lateral sclerosis, and schizophrenia.^16^ The fine regulation of genes associated with pathologies through miRNAs could be an important mechanism to maintain neuronal homeostasis;^17^ in particular, several studies show that the misregulation and alterations of specific miRNAs could contribute to the etiopathogenesis of AD.^18,19^ Among these, miRNA-29a, miRNA-29b, and miRNA-29c are a class of miRNAs known to regulate brain β-secretase cleaving enzyme 1 (BACE1) expression and the pathogenesis of AD,^9,20^ and in particular miRNA-29a seems to exert a “protective” role in AD.^21^

It has been reported that, in brains of AD patients, miRNA-29a decreases, leading to increased BACE1 gene expression. BACE1 is the rate-limiting enzyme responsible for Aβ production in the brain, and increased BACE1 expression is thought to be a risk factor for sporadic AD.^22^ Many studies aimed at testing the effect of small molecule inhibitors on reducing BACE1 activity are ongoing and may show promising results in view of the discovery of a disease-modulating treatment;^23^ our attempt to identify miRNA-29a as a possible AD biomarker is therefore well supported by the current efforts of AD research. Additionally, the miRNA-29 family is reported to regulate DNA methyltransferases 3A and 3B, and this can cause cell death. Thus, decreasing miRNA-29a in brain would lead to increased Aβ generation and DNA methylation, thereby becoming a burden for aged neurons,^24^ and previous reports indicate that plasma miRNA-29a levels were unchanged in AD patients versus controls.^25^ About the detection of miRNAs, conventional analysis usually includes Northern blotting,^26^ microarrays,^27^ and quantitative reverse transcription PCR (RT-qPCR).^28,29^ However, these techniques are characterized by several time-consuming steps including isolation of target, gel denaturation, transfer to solid support for the blotting, and the use of fluorescence readers and proprietary instruments and software in the case of microarrays. These approaches are also characterized by expensive costs of the kits, considering an average cost between $700 and $1000 (excluding the cost of instruments). However, Northern blotting is today almost completely replaced by PCR in targeted assays (i.e., assays aimed at revealing the expression of specific miRNAs). Microarrays, on the other hand, remain the gold standard to investigate the expression pattern of the complete miRNome, requiring PCR confirmation for selected sequences. The biosensor described here (possibly adapted to other circulating AD-related miRNAs) offers the possibility of a rapid and cheap assay, not involving highly specialized personnel and expensive instruments as in the RT-qPCR assay. In particular, the adoption of RT-qPCR is also not suitable for a portable screening tool, considering RNA is first transcribed into complementary DNA by reverse transcriptase and the use of primers and proprietary instruments is necessary for the quantification.

Due to that, there is a still a need to develop easy-to-use, low-cost, sensitive methods to facilitate the detection of AD and that require smaller amounts of sample to minimize the extraction procedures performed on patients, as in the case of biosensors. Among the different classes of biosensors, the electrochemical ones have their main advantages of being miniaturized and unaffected by colored matrixes (which represent the first limitation for colorimetric-based methods).^30^ Regarding the detection of miRNA sequences, various electrochemical examples involving different strategies have been reported in the literature, with application toward various diseases and conditions, i.e., cancer, osteoporosis, obesity, infections, etc.^31,32^ There are a limited number of examples of the electrochemical sensing of miRNAs implicated in the pathogenesis of AD, such as miRNA-34a, miRNA-137, miRNA-146a, and miRNA-101. In the work carried out by Congur et al.,^35^ a graphene oxide (GO) based single-use electrochemical biosensor was developed for the sensitive and selective impedimetric detection of miRNA-34a as biomarker of Alzheimer’s disease^33^ and various types of cancer,^34^ using a pencil graphite electrode (PGE) and an miRNA-34a specific DNA probe as the recognition element. The measurement was performed through electrochemical impedance spectroscopy (EIS), obtaining a limit of detection of ca. 70 nM in diluted serum.^35^ Another miRNA used as a suitable biomarker for electrochemical sensing was miRNA-137, detected in the femtomolar range by Azimzadeh and colleagues.^36^ They developed an ultrasensitive electrochemical nanobiosensor to quantify serum miRNA-137 on a screen-printed carbon electrode (SPCE) modified with electrochemically reduced graphene oxide (ERGO) and gold nanowires (AuNWs), using doxorubicin (Dox) as an intercalated label. The device showed a limit of detection of 1.7 fM with high specificity toward the target oligo also in serum samples, revealing potential clinical applications for the early detection of AD.^36^ The same miRNA target was also recently detected by Chang at al. with their graphene oxide constructed triangular electrodes specific to detect miRNA-137 using the complementary sequence as the recognition probe.^37^ To enhance the immobilization of capturing miRNA-137, gold nanostars (GNS) were conjugated with the immobilized capture probe, leading to a 10 fM detection limit. Song and co-workers developed a multiplatform for the determination of multiple Alzheimer’s biomarkers, including tau, ApoE4, amyloid β, and miRNA-101, exploiting the advantage of minipillar-based architectures.^38^ It should be noted that each biosensor was built onto a different nanopillar, and all the nanopillars were electrodeposited with of gold nanodendrites. Regarding the detection of miRNA-101, a ferrocene-modified DNA probe was used to develop a signal-off platform, capable of detecting the target down to ca. 90 pM.^38^

However, to the best of our knowledge, there are no reports on electrochemical methods for the quantification of miRNA-29 for AD early detection. In this work our attention has been focused on miRNA-29, because of its role in the pathogenesis of AD. The choice of the target is novel in the field of electrochemical printed devices, considering the other existing approaches that have been developed using other methods/procedures such as surface enhanced Raman scattering,^39^ plasmonic colorimetric strategy based on the hybridization chain reaction,^40^ and a Förster resonance electron transfer based biosensor.^41^ Most of the methods that have been reported are built on mechanisms requiring a multistep and/or sophisticated setup for users. The developed electrochemical platform was applied toward the detection of miRNA-29a in both buffer and serum matrix, highlighting satisfactory selectivity and low detection limit at the level of low nanomolar, respectively, 0.15 and 0.2 nM. These results demonstrated a very satisfactory sensitivity and simplicity of operations compared with similar architectures for the detection of miRNAs in biological matrixes: a paper-based peptide nucleic acid based biosensor was used to detect miRNA-492, a pancreatic ductal adenocarcinoma biomarker, down to 6 nM,^31^ and recently a AuNP superlattice and conductive polypyrrole on a glassy carbon electrode, in the presence of toluidine blue as the redox probe, allowed a detection of miRNA-21 in the subnanomolar range, with a total duration of 3.5 h (compared with the ca. 3 h of the present approach).^42^

## Materials and Methods

### Reagents, Instruments, and Screen-Printing

All this information is reported in the Supporting Information.

### Synthesis of Gold Nanoparticles (AuNPs) Dispersion

AuNPs have been obtained as described in the literature,^44^ and the procedure is reported in the Supporting Information. The AuNPs have been characterized through SEM, DLS, and EDS experiments. DLS measurements revealed a monodispersed AuNP suspension as reported in Figure S1 with an average diameter of 196.2 ± 20.65 nm and a polydispersity index (PDI) of 0.13 ± 0.07, highlighting the synthesis of uniform and monodisperse particles.

### Preparation of the Strip Specific for miRNA-29a Sensing

In order to customize the area of the working electrode, 8 μL of AuNPs was drop casted onto the working electrode and, after drying, the probe was immobilized following a protocol reported in the literature.^43^ Another effective approach to modify electrochemical systems with AuNPs is electrodeposition.^44,45^ However, even if the performance is not significantly affected by the selected approach,^46^ our choice to exploit drop casting has been a consequence of the possibility to modify multiple electrodes at the same time toward a mass-scale production. The anti-miRNA-29a DNA sequence was used as probe to be attached onto the working electrode surface of the AuNP screen-printed electrode (AuNP-SPE). The probe was customized with C6-SH at the 5′-end (allowing covalent binding onto AuNPs) and with methylene blue (MB) at the 3′-end (allowing electron transfer at the electrode). The first step was represented by the reduction of the 100 μM anti-miRNA-29a DNA probe (50 mM phosphate buffer containing 1 M NaCl and 10 mM MgCl_2_ at pH 7) in the presence of 10 mM tris(2-carboxyethyl)phosphine hydrochloride) (TCEP) for 1 h. TCEP is necessary to reduce the disulfide bond of the probe prior to being covalently attached on top of the AuNPs. The resulting solution was then diluted to the selected concentration (in the nanomolar range) to be immobilized onto the AuNP-SPE. Subsequently, 20 μL of the probe was placed onto the working electrode area for 1 h at RT in a humid chamber. SPEs were gently washed with distilled water and incubated in a humid chamber with 20 μL of 2 mM 6-mercapto-1-hexanol for 1.5 h to passivate the empty spaces onto the working electrode. The last step was washing away with distilled water the unbound thiol from the SPEs, and the measurement was performed by adding the working buffer solution.

### Microscale Thermophoresis (MST) Experiments

All MST measurements were performed on a Monolith NT.115 instrument (NanoTemper Technologies, Munich, Germany) with the use of Standard Treated Capillaries from the supplier, and the procedure is reported in the Supporting Information.

### Measurement of miRNA Targets

**Figure 1:**
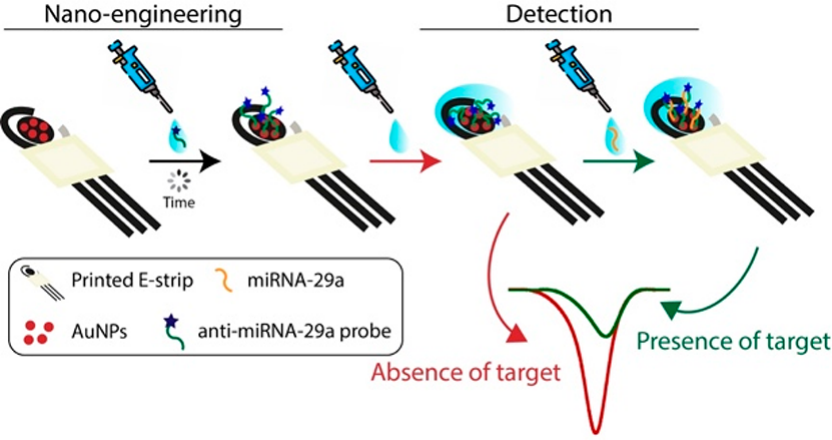
Schematic representation for the development of the electrochemical biosensor for the detection of miRNA-29 and the expected current signal in the absence and presence of target.

For the measurement of miRNA targets, eight SPEs were inserted into the multiplexer connected to the portable potentiostat. To the electrochemical cells 100 μL of working buffer, 50 mM phosphate buffer containing 1 M NaCl and 10 mM MgCl_2_ at pH 7, was added. The measurements were carried out after 30 min with respect to the addition of sample. For all the optimization experiments and quantification curves, the signal change (%) = ((*I*_0_ – *I*_target_)/*I*_0_) × 100 (where *I*_0_ and *I*_target_ are the current values obtained in the absence and presence of target, respectively) was visualized against the concentration of the target analyzed and the measure in the absence of target was used as the background current, as shown in Figure 1.

The formation of a rigid probe/target complex reduced the electron transfer at the electrode and resulted in a signal-off platform in which, as the target concentration increases, the signal change percent increases until a stationary phase is reached.^47,48^

## Results and Discussion

### MB-Probe/miRNA-29a Binding Characterization in Solution

**Figure 2:**
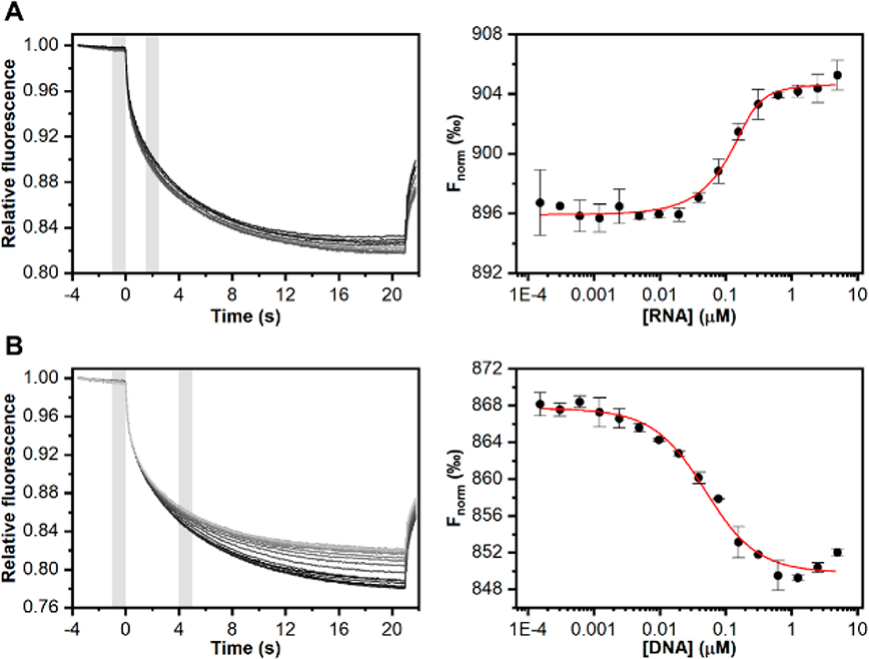
MST measurements for the hybridization of the labeled oligonucleotide with (A) RNA and (B) DNA complementary target sequences in solution. (left) Fluorescence time traces recorded by incubating increasing concentrations of the target sequences with the labeled oligonucleotide. (right) Corresponding binding curves. Error bars represent standard error of the mean.

Prior to starting with the development of the electrochemical strip for miRNA-29a detection, the interaction between the DNA probe labeled with methylene blue (MB) at the 3′-end and the DNA and RNA carrying the complementary sequences was assessed by microscale thermophoresis (MST), obtaining quantitative information on the affinity of the DNA/DNA duplex and the DNA/RNA heteroduplex in solution. Indeed, MST is a well-established and versatile tool that uses fluorescence detection to monitor the directed diffusion (thermophoresis) of biomolecules along a temperature gradient for the quantitative analysis of bimolecular binding events, including oligonucleotide hybridization reactions.^49^ To this purpose, the hybridization reactions were analyzed by titrating 200 nM labeled DNA probe with increasing concentrations of the complementary sequences (Figure 2). Fitting of the relative change of thermophoresis upon DNA hybridization yielded equilibrium dissociation constant (*K*_d_) values of 25 and 40 nM for RNA and DNA, respectively. These data indicate that the labeled DNA probe has a higher affinity for RNA than for DNA. This is in agreement with the literature data in which it is reported that the DNA/RNA heteroduplex formation process is thermodynamically favored since the DNA/RNA hybrid interaction is much stronger and more stable than that of DNA/DNA duplexes.^50,51^

### Evaluation of the Experimental Setup

**Figure 3:**
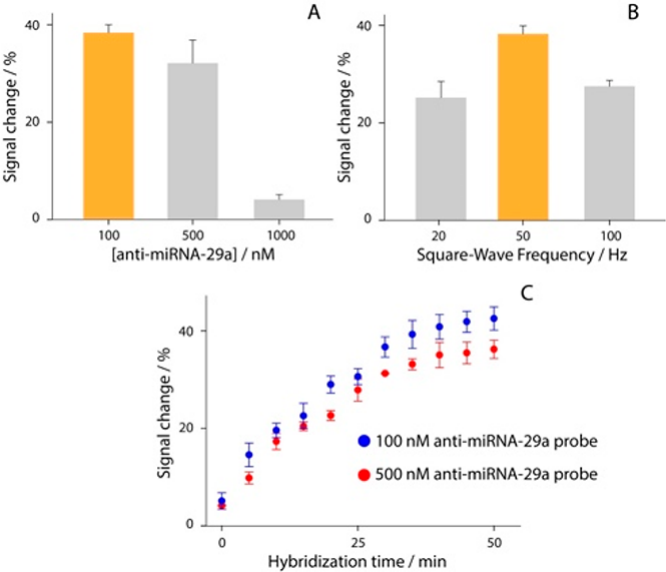
(A) Study of anti-miRNA-29a specific probe density at 100, 250, and 1000 nM in the presence of 80 nM target. (B) Study of the square-wave frequency between 20 and 100 Hz in the presence of 50 nM target and using 100 nM as the starting solution for probe immobilization. (C) Study of hybridization kinetics up to 50 min using two probe densities: 100 (blue dots) and 500 nM (red dots).

The development of a portable electrochemical device for miRNA detection is dependent on the choice of the optimal experimental parameters, considering the immobilized probe density, the signal-to-noise ratio, the duration of testing, and the electrochemical settings. An important step in the fabrication of the device is represented by the density of the immobilized probe and the homogeneity of the surface. In general, for surface-bound probes that are modified with redox mediator, the higher the probe density is, the higher the current recorded is. However, the affinity for the target is lower when the density of the immobilized probe is high, due to the diffusion limitation of the target in reaching the probe. This should be considered the optimal compromise between signal change and signal-to-noise ratio, as shown in Figure 3.

The study was first focused on evaluating the response of platforms when 100, 500, and 1000 nM anti-miRNA-29a probe were immobilized onto the surface, in the presence of 80 nM miRNA-29a target. As displayed, the 100 nM probe solution allowed obtaining of the highest signal variation of ca. 40% with a repeatability (calculated as the relative standard deviation) of 7% when compared with other densities tested. This concentration was selected for the manufacturing of the strips, due to the satisfactory compromise between signal change and detectable current. Subsequently, the effect of the square-wave frequency was evaluated, in a range between 20 and 100 Hz. For these experiments, 50 mM phosphate buffer containing 1 M NaCl and 10 mM MgCl_2_ at pH 7 was used, and 20, 50, and 100 Hz were interrogated in the presence of 50 nM target. As shown in Figure 3B, 50 Hz represented a good compromise in terms of sensitivity and repeatability (based on four replicates), and it was chosen to further develop the work. The last investigated parameter was consistent in the binding time to form the probe–target duplex. Considering the final development of a point-of-care test for miRNA-29a detection, the binding process between probe and target represents a significant step, having a significant impact on the duration of the analysis. Specifically, the time requested to form the probe–target complex is strictly dependent on the nature and length of sequences and on the probe + target ↔ probe–target equilibrium, and in the literature it is reported that the formation of this kind of structure for ca. 20 bp targets requires about 15–20 min.^52^ Thus, after having covered the testing area of the strip with a fixed concentration of target, namely 50 nM, the probe–target interaction time was carefully evaluated. The study was performed in a time range up to 50 min, as shown in Figure 3C. Two probe densities were compared, 100 and 500 nM, demonstrating that there was a slight difference among the two curves and a time of 30 min was consistent with a satisfactory compromise in term of sensitivity, repeatability, and time of analysis, since the signal was not significantly affected by longer times.

### Analytical Features

**Figure 4:**
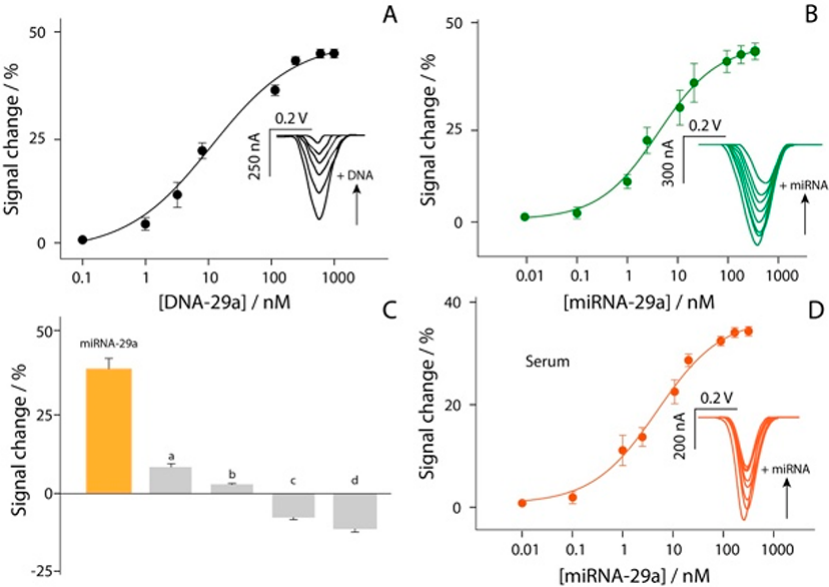
Calibration curve and SWV curves obtained in buffer solution testing different concentrations of (A) DNA-29a target from 0.1 to 1000 nM and (B) miRNA-29a target from 0.01 to 500 nM. (C) Selectivity studies in buffer solutions, comparing the signal intensities obtained in the presence of 10 nM miRNA-29a (orange bar) and in the presence of not-complementary RNA single strands (a, b) and DNA single strands (c, d). (D) Calibration curve and SWV curves obtained in 90%-diluted human serum testing different concentrations of miRNA-29a target from 0.01 to 500 nM. All the experiments have been carried out in triplicate, and the experimental conditions are those reported in the caption of Figure 3.

Once all main experimental features were evaluated, the analytical performance of the printed platform was evaluated in the presence of increasing concentrations of the target ranging from 0.1 to 1000 nM, as reported in Figure 4.

The experiments were performed with two different targets: in addition to the miRNA-29a sequence, the DNA-based target was also analyzed, to better characterize the immobilized recognition architecture. For all the experiments, the optimized settings were adopted, and a semilogarithmic sigmoidal trend was observed between the signal change and the concentration of target (expressed in log scale). The measurements in buffer solution were characterized by a sigmoidal trend. As shown in Figure 4A,B, each calibration point displayed in the curves is obtained as the result of four separate devices. The correlations of the two platforms were equal to 0.992 and 0.983 for DNA-based and miRNA-based targets, respectively. In addition, the detection limit was calculated as the concentration corresponding to a 10% variation of the signal, and it has been approximated to ca. 1.0 and 0.15 nM for DNA-based and miRNA-based targets, respectively. A correlation described by *R* = 0.988 was obtained comparing the herein reported miRNA-29a detection with a UV–vis reading selecting 260 nm as the wavelength. The selectivity of the platform was evaluated in the presence of four interferents, namely two miRNA sequences and two DNA sequences (Supporting Information), as shown in Figure 4C.

The study was carried out using 10 nM concentration of each species, and as reported in Figure 4C, only negligible interferences were observed (up to 10%), thus confirming the satisfactory selectivity of the sensing platform. Subsequently, the electrochemical strip was applied toward the determination of miRNA-29a in serum samples. The matrix effect was evaluated, as shown in Figure S2, and 90%-diluted human serum resulted as the optimal compromise to conduct detection. It was spiked with various concentrations of target up to 100 nM (Figure 4D). Even in this case a good sigmoidal correlation was obtained, with an *R*^2^ of 0.991, and the detection limit was calculated equal to ca. 0.2 nM. These features confirm the possibility of the present electrochemical platform to be adopted as a sensing tool for the management of AD at the point-of-care.

## Conclusion

In this work the optimization and development of a printed electrochemical tool for miRNA-29a recognition were carried out. miRNA-29a was chosen due to its promising value toward the management of Alzheimer’s disease at the point-of-care. The screen-printed platform was modified with gold nanoparticles, and subsequently a DNA probe was immobilized on top of the electrode. Square-wave voltammetry was used as the quantification method. The probe was covalently modified with methylene blue (MB) as the redox tag allowing the electrochemical transduction. All the parameters were optimized, including the density of the probe, the square-wave frequency, time of probe–target hybridization, and the binding constant. The designed architecture was able to correlate the decrease of the MB-based current with the increase of target concentration, namely signal off, as a consequence of a conformational change that led to a more rigid probe–target structure with respect to the flexible probe, thus limiting the electron transfer between MB and the electrode. Both microscale thermophoresis and electrochemical characterization have demonstrated the satisfactory application of a DNA-based probe for the recognition of miRNA target. After consideration of the experimental parameters to be exploited, the electrochemical strip was able to detect miRNA-29a down to ca. 0.15 and 0.2 nM in a few microliters of standard solution and spiked serum, respectively. The platform displayed satisfying output also in the presence of interfering species. If circulating biomarkers are validated, these kinds of platforms will be capable of giving quick answers to patients, reducing the time gap between the diagnosis and treatment of a devastating and silent disease such as Alzheimer’s. The continuous collaboration of complementary disciplines such as decentralized analytical chemistry, bioengineering, physical chemistry, and neuroscience can lead to tremendous improvements for the entire range of neurodegenerative diseases. The results demonstrated the suitability of this approach in terms of ease of use by unskilled personnel, time of production, sensitivity, and applicability. The adoption of polyester-based substrates for manufacturing electrochemical strips compared to paper-based ones allows the creation of more robust platforms to be applied in a decentralized context. The entire platform was conceived as a disposable sensor, able to give rapid answers to patients, reducing the time gap between diagnosis and treatment.

## Supporting Information Available

Reagents and instruments utilized; screen-printing process; AuNPs synthesis; microscale thermophoresis; characterization of AuNPs and matrix effect evaluation in serum (PDF)

## References

[ref1] Lane C. A.; Hardy J.; Schott J. M. Alzheimer’s Disease. European Journal of Neurology 2018, 25 (1), 59–70. 10.1111/ene.13439. 28872215

[ref2] Silvestro S.; Bramanti P.; Mazzon E. Role of MiRNAs in Alzheimer’s Disease and Possible Fields of Application. International Journal of Molecular Sciences 2019, 20 (16), 3979 10.3390/ijms20163979. PMC6720959 31443326

[ref3] Allone C.; Lo Buono V.; Corallo F.; Bonanno L.; Palmeri R.; Di Lorenzo G.; Marra A.; Bramanti P.; Marino S. Cognitive Impairment in Parkinson’s Disease, Alzheimer’s Dementia, and Vascular Dementia: The Role of the Clock-Drawing Test. Psychogeriatrics 2018, 18 (2), 123–131. 10.1111/psyg.12294. 29417704

[ref4] Müller U. C.; Deller T.; Korte M. Not Just Amyloid: Physiological Functions of the Amyloid Precursor Protein Family. Nat. Rev. Neurosci 2017, 18 (5), 281–298. 10.1038/nrn.2017.29. 28360418

[ref5] Norton S.; Matthews F. E.; Barnes D. E.; Yaffe K.; Brayne C. Potential for Primary Prevention of Alzheimer’s Disease: An Analysis of Population-Based Data. Lancet Neurology 2014, 13 (8), 788–794. 10.1016/S1474-4422(14)70136-X. 25030513

[ref6] Hardy J. A.; Higgins G. A. Alzheimer’s Disease: The Amyloid Cascade Hypothesis. Science 1992, 256 (5054), 184–185. 10.1126/science.1566067. 1566067

[ref7] Zhang Y.; Thompson R.; Zhang H.; Xu H. APP Processing in Alzheimer’s Disease. Molecular Brain 2011, 4 (1), 3 10.1186/1756-6606-4-3. 21214928 PMC3022812

[ref8] Bekris L. M.; Yu C.-E.; Bird T. D.; Tsuang D. W. Genetics of Alzheimer Disease. J. Geriatr Psychiatry Neurol 2010, 23 (4), 213–227. 10.1177/0891988710383571. 21045163 PMC3044597

[ref9] Hébert S. S.; Horré K.; Nicolaï L.; Papadopoulou A. S.; Mandemakers W.; Silahtaroglu A. N.; Kauppinen S.; Delacourte A.; De Strooper B. Loss of MicroRNA Cluster MiR-29a/b-1 in Sporadic Alzheimer’s Disease Correlates with Increased BACE1/β-Secretase Expression. Proc. Natl. Acad. Sci. U. S. A. 2008, 105 (17), 6415–6420. 10.1073/pnas.0710263105. 18434550 PMC2359789

[ref10] Henriksen K.; O’Bryant S. E.; Hampel H.; Trojanowski J. Q.; Montine T. J.; Jeromin A.; Blennow K.; Lönneborg A.; Wyss-Coray T.; Soares H.; Bazenet C.; Sjögren M.; Hu W.; Lovestone S.; Karsdal M. A.; Weiner M. W. The Future of Blood-Based Biomarkers for Alzheimer’s Disease. Alzheimers Dement 2014, 10 (1), 115–131. 10.1016/j.jalz.2013.01.013. 23850333 PMC4128378

[ref11] Morris J. C. The Clinical Dementia Rating (CDR): Current Version and Scoring Rules. Neurology 1993, 43 (11), 2412 10.1212/WNL.43.11.2412-a. 8232972

[ref12] Scheltens P. Imaging in Alzheimer’s Disease. Dialogues Clin Neurosci 2009, 11 (2), 191–199. 10.31887/DCNS.2009.11.2/pscheltens. 19585954 PMC3181915

[ref13] Li R.; Rui G.; Chen W.; Li S.; Schulz P. E.; Zhang Y. Early Detection of Alzheimer’s Disease Using Non-Invasive Near-Infrared Spectroscopy. Front Aging Neurosci 2018, 10, 366 10.3389/fnagi.2018.00366. 30473662 PMC6237862

[ref14] Fagan A. M.; Perrin R. J. Upcoming Candidate Cerebrospinal Fluid Biomarkers of Alzheimer’s Disease. Biomark Med. 2012, 6 (4), 455–476. 10.2217/bmm.12.42. 22917147 PMC3477809

[ref15] Siedlecki-Wullich D.; Miñano-Molina A. J.; Rodríguez-Álvarez J. MicroRNAs as Early Biomarkers of Alzheimer’s Disease: A Synaptic Perspective. Cells 2021, 10 (1), 113 10.3390/cells10010113. 33435363 PMC7827653

[ref16] Reddy P. H.; Tonk S.; Kumar S.; Vijayan M.; Kandimalla R.; Kuruva C. S.; Reddy A. P. A Critical Evaluation of Neuroprotective and Neurodegenerative MicroRNAs in Alzheimer’s Disease. Biochem. Biophys. Res. Commun. 2017, 483 (4), 1156–1165. 10.1016/j.bbrc.2016.08.067. 27524239 PMC5343756

[ref17] Schratt G. MicroRNAs at the Synapse. Nat. Rev. Neurosci 2009, 10 (12), 842–849. 10.1038/nrn2763. 19888283

[ref18] Lukiw W. J. Micro-RNA Speciation in Fetal, Adult and Alzheimer’s Disease Hippocampus. NeuroReport 2007, 18 (3), 297–300. 10.1097/WNR.0b013e3280148e8b. 17314675

[ref19] Guedes J. R.; Santana I.; Cunha C.; Duro D.; Almeida M. R.; Cardoso A. M.; de Lima M. C. P.; Cardoso A. L. MicroRNA Deregulation and Chemotaxis and Phagocytosis Impairment in Alzheimer’s Disease. Alzheimers Dementia: Diagnosis, Assessment Disease Monitoring 2016, 3 (1), 7–17. 10.1016/j.dadm.2015.11.004. PMC4879648 27239545

[ref20] Zong Y.; Yu P.; Cheng H.; Wang H.; Wang X.; Liang C.; Zhu H.; Qin Y.; Qin C. MiR-29c Regulates NAV3 Protein Expression in a Transgenic Mouse Model of Alzheimer’s Disease. Brain Res. 2015, 1624, 95–102. 10.1016/j.brainres.2015.07.022. 26212654

[ref21] Müller M.; Jäkel L.; Bruinsma I. B.; Claassen J. A.; Kuiperij H. B.; Verbeek M. M. MicroRNA-29a Is a Candidate Biomarker for Alzheimer’s Disease in Cell-Free Cerebrospinal Fluid. Mol. Neurobiol 2016, 53 (5), 2894–2899. 10.1007/s12035-015-9156-8. 25895659 PMC4902829

[ref22] Hampel H.; Vassar R.; De Strooper B.; Hardy J.; Willem M.; Singh N.; Zhou J.; Yan R.; Vanmechelen E.; De Vos A.; Nisticò R.; Corbo M.; Imbimbo B. P.; Streffer J.; Voytyuk I.; Timmers M.; Monfared A. A. T.; Irizarry M.; Albala B.; Koyama A.; Watanabe N.; Kimura T.; Yarenis L.; Lista S.; Kramer L.; Vergallo A. The β-Secretase BACE1 in Alzheimer’s Disease. Biol. Psychiatry 2021, 89 (8), 745–756. 10.1016/j.biopsych.2020.02.001. 32223911 PMC7533042

[ref23] McDade E.; Voytyuk I.; Aisen P.; Bateman R. J.; Carrillo M. C.; De Strooper B.; Haass C.; Reiman E. M.; Sperling R.; Tariot P. N.; Yan R.; Masters C. L.; Vassar R.; Lichtenthaler S. F. The case for low-level BACE1 inhibition for the prevention of Alzheimer disease. Nat. Rev. Neurol 2021, 17 (11), 703–714. 10.1038/s41582-021-00545-1. 34548654

[ref24] Fuso A.; Raia T.; Orticello M.; Lucarelli M. The Complex Interplay between DNA Methylation and MiRNAs in Gene Expression Regulation. Biochimie 2020, 173, 12–16. 10.1016/j.biochi.2020.02.006. 32061806

[ref25] Kiko T.; Nakagawa K.; Tsuduki T.; Furukawa K.; Arai H.; Miyazawa T. MicroRNAs in Plasma and Cerebrospinal Fluid as Potential Markers for Alzheimer’s Disease. J. Alzheimers Dis 2014, 39 (2), 253–259. 10.3233/JAD-130932. 24157723

[ref26] Várallyay E.; Burgyán J.; Havelda Z. MicroRNA Detection by Northern Blotting Using Locked Nucleic Acid Probes. Nat. Protoc 2008, 3 (2), 190–196. 10.1038/nprot.2007.528. 18274520

[ref27] Li W.; Ruan K. MicroRNA Detection by Microarray. Anal Bioanal Chem. 2009, 394 (4), 1117–1124. 10.1007/s00216-008-2570-2. 19132354

[ref28] Mohammadi-Yeganeh S.; Paryan M.; Mirab Samiee S.; Soleimani M.; Arefian E.; Azadmanesh K.; Mostafavi E.; Mahdian R.; Karimipoor M. Development of a Robust, Low Cost Stem-Loop Real-Time Quantification PCR Technique for MiRNA Expression Analysis. Mol. Biol. Rep 2013, 40 (5), 3665–3674. 10.1007/s11033-012-2442-x. 23307300

[ref29] Niu Y.; Zhang L.; Qiu H.; Wu Y.; Wang Z.; Zai Y.; Liu L.; Qu J.; Kang K.; Gou D. An Improved Method for Detecting Circulating MicroRNAs with S-Poly(T) Plus Real-Time PCR. Sci. Rep 2015, 5, 15100 10.1038/srep15100. 26459910 PMC4602224

[ref30] Singh S.; Wang J.; Cinti S. Review—An Overview on Recent Progress in Screen-Printed Electroanalytical (Bio)Sensors. ECS Sens. Plus 2022, 1 (2), 023401 10.1149/2754-2726/ac70e2.

[ref31] Moccia M.; Caratelli V.; Cinti S.; Pede B.; Avitabile C.; Saviano M.; Imbriani A. L.; Moscone D.; Arduini F. Paper-Based Electrochemical Peptide Nucleic Acid (PNA) Biosensor for Detection of MiRNA-492: A Pancreatic Ductal Adenocarcinoma Biomarker. Biosens. Bioelectron. 2020, 165, 112371 10.1016/j.bios.2020.112371. 32729503

[ref32] Sher M.; Faheem A.; Asghar W.; Cinti S. Nano-Engineered Screen-Printed Electrodes: A Dynamic Tool for Detection of Viruses. TrAC Trends in Analytical Chemistry 2021, 143, 116374 10.1016/j.trac.2021.116374. 34177011 PMC8215883

[ref33] Cogswell J. P.; Ward J.; Taylor I. A.; Waters M.; Shi Y.; Cannon B.; Kelnar K.; Kemppainen J.; Brown D.; Chen C.; Prinjha R. K.; Richardson J. C.; Saunders A. M.; Roses A. D.; Richards C. A. Identification of MiRNA Changes in Alzheimer’s Disease Brain and CSF Yields Putative Biomarkers and Insights into Disease Pathways. Journal of Alzheimers Disease 2008, 14 (1), 27–41. 10.3233/JAD-2008-14103. 18525125

[ref34] Chalanqui M. J.; O’Doherty M.; Dunne N. J.; McCarthy H. O. MiRNA 34a: A Therapeutic Target for Castration-Resistant Prostate Cancer. Expert Opinion on Therapeutic Targets 2016, 20 (9), 1075–1085. 10.1517/14728222.2016.1162294. 26942553

[ref35] Congur G.; Eksin E.; Erdem A. Impedimetric Detection of MiRNA-34a Using Graphene Oxide Modified Chemically Activated Graphite Electrodes. Sensors and Actuators A: Physical 2018, 279, 493–500. 10.1016/j.sna.2018.06.026.

[ref36] Azimzadeh M.; Nasirizadeh N.; Rahaie M.; Naderi-Manesh H. Early Detection of Alzheimer’s Disease Using a Biosensor Based on Electrochemically-Reduced Graphene Oxide and Gold Nanowires for the Quantification of Serum MicroRNA-137. RSC Adv. 2017, 7 (88), 55709–55719. 10.1039/C7RA09767K.

[ref37] Chang W.; Zhao J.; Liu L.; Xing X.; Zhang C.; Meng H.; Gopinath S. C. B.; Liu Y. Graphene Oxide-Gold Star Construct on Triangular Electrodes for Alzheimer’s Disease Identification. Journal of Analytical Methods in Chemistry 2021, 2021, 6661799 10.1155/2021/6661799. 33688447 PMC7920714

[ref38] Song Y.; Xu T.; Zhu Q.; Zhang X. Integrated Individually Electrochemical Array for Simultaneously Detecting Multiple Alzheimer’s Biomarkers. Biosens. Bioelectron. 2020, 162, 112253 10.1016/j.bios.2020.112253. 32392158

[ref39] Mabbott S.; Fernandes S. C.; Schechinger M.; Cote G. L.; Faulds K.; Mace C. R.; Graham D. Detection of cardiovascular disease associated miR-29a using paper-based microfluidics and surface enhanced Raman scattering. Analyst 2020, 145, 983–991. 10.1039/C9AN01748H. 31829323

[ref40] Miao J.; Wang J.; Guo J.; Gao H.; Han K.; Jiang C.; Miao P. A plasmonic colorimetric strategy for visual miRNA detection based on hybridization chain reaction. Sci. Rep. 2016, 6, 1–7. 10.1038/srep32219. 27534372 PMC4989231

[ref41] Kim H. I.; Yim D.; Jeon S. J.; Kang T. W.; Hwang I. J.; Lee S.; et al. Modulation of oligonucleotide-binding dynamics on WS2 nanosheet interfaces for detection of Alzheimer’s disease biomarkers. Biosens. Bioelectron. 2020, 165, 112401 10.1016/j.bios.2020.112401. 32729521

[ref42] Tian L.; Qian K.; Qi J.; Liu Q.; Yao C.; Song W.; Wang Y. Gold nanoparticles superlattices assembly for electrochemical biosensor detection of microRNA-21. Biosens. Bioelectron. 2018, 99, 564–570. 10.1016/j.bios.2017.08.035. 28826000

[ref43] Xiao Y.; Lai R. Y.; Plaxco K. W. Preparation of Electrode-Immobilized, Redox-Modified Oligonucleotides for Electrochemical DNA and Aptamer-Based Sensing. Nat. Protoc 2007, 2 (11), 2875–2880. 10.1038/nprot.2007.413. 18007622

[ref44] Florea A.; Taleat Z.; Cristea C.; Mazloum-Ardakani M.; Săndulescu R. Label free MUC1 aptasensors based on electrodeposition of gold nanoparticles on screen printed electrodes. Electrochemistry communications 2013, 33, 127–130. 10.1016/j.elecom.2013.05.008.

[ref45] Hezard T.; Fajerwerg K.; Evrard D.; Collière V.; Behra P.; Gros P. Influence of the gold nanoparticles electrodeposition method on Hg (II) trace electrochemical detection. Electrochim. Acta 2012, 73, 15–22. 10.1016/j.electacta.2011.10.101.

[ref46] Ortone V.; Matino L.; Santoro F.; Cinti S. Merging office/filter paper-based tools for pre-concentring and detecting heavy metals in drinking water. Chem. Commun. 2021, 57, 7100–7103. 10.1039/D1CC02481G. 34169301

[ref47] Cinti S.; Cinotti G.; Parolo C.; Nguyen E. P.; Caratelli V.; Moscone D.; Arduini F.; Merkoci A. Experimental Comparison in Sensing Breast Cancer Mutations by Signal ON and Signal OFF Paper-Based Electroanalytical Strips. Anal. Chem. 2020, 92 (2), 1674–1679. 10.1021/acs.analchem.9b02560. 31876409

[ref48] Cinti S.; Proietti E.; Casotto F.; Moscone D.; Arduini F. Paper-Based Strips for the Electrochemical Detection of Single and Double Stranded DNA. Anal. Chem. 2018, 90 (22), 13680–13686. 10.1021/acs.analchem.8b04052. 30338973

[ref49] Jerabek-Willemsen M.; André T.; Wanner R.; Roth H. M.; Duhr S.; Baaske P.; Breitsprecher D. MicroScale Thermophoresis: Interaction Analysis and Beyond. J. Mol. Struct. 2014, 1077, 101–113. 10.1016/j.molstruc.2014.03.009.

[ref50] Chein Y.-H.; Davidson N. RNA:DNA Hybrids Are More Stable than DNA:DNA Duplexes in Concentrated Perchlorate and Trichloroacetate Solutions. Nucleic Acids Res. 1978, 5 (5), 1627–1637. 10.1093/nar/5.5.1627. 208059 PMC342109

[ref51] Sugimoto N.; Nakano S.; Katoh M.; Matsumura A.; Nakamuta H.; Ohmichi T.; Yoneyama M.; Sasaki M. Thermodynamic Parameters To Predict Stability of RNA/DNA Hybrid Duplexes. Biochemistry 1995, 34 (35), 11211–11216. 10.1021/bi00035a029. 7545436

[ref52] Patterson A.; Caprio F.; Vallée-Bélisle A.; Moscone D.; Plaxco K. W.; Palleschi G.; Ricci F. Using Triplex-Forming Oligonucleotide Probes for the Reagentless, Electrochemical Detection of Double-Stranded DNA. Anal. Chem. 2010, 82 (21), 9109–9115. 10.1021/ac1024528. 20936782 PMC3134121

