# Supplementary material for: Printed Electrochemical Strip for the Detection of miRNA-29a: A Possible Biomarker Related to Alzheimer’s Disease
Source: Anal Chem. 2022 Nov 1;94(45):15558–63. doi: 10.1021/acs.analchem.2c03542 (PMC9670028; doi:10.1021/acs.analchem.2c03542)
Supplement: Supplementary file 1 — ac2c03542_si_001.pdf [file ac2c03542_si_001.pdf]

Printed electrochemical strip for the detection of miRNA-29a: a possible biomarker related to Alzheimer's disease

Antonella Miglione[1], Ada Raucci[1], Jussara Amato[1], Simona Marzano[1], Bruno Pagano[1], Tiziana Raia[2], Marco Lucarelli[2,3], Andrea Fuso[2], Stefano Cinti[1,4,*]

[1]Department of Pharmacy, University of Naples "Federico II", Via Domenico Montesano 49, 80131 Naples, Italy.
[2]Department of Experimental Medicine, Sapienza University of Rome, Viale Regina Elena 324, 00161 Rome, Italy
[3]Pasteur Institute Cenci Bolognetti Foundation, Sapienza University of Rome, Rome, Italy
[4]BAT Center - Interuniversity Center for Studies on Bioinspired Agro-Environmental Technology, University of Naples "Federico II", 80055 Naples, Italy

* Corresponding author: stefano.cinti@unina.it

Abstract
Here are reported the information regarding the reagents and instruments that have been utilized, the screen-printing process, AuNPs synthesis, Microscale thermophoresis, characterization of the AuNPs, sequences used for selectivity studies and the matrix effect evaluation in serum have been reported.

Reagents and equipment

Chloroauric acid (HAuCl4), sodium borohydride, sodium citrate, sodium chloride, sodium dihydrogen phosphate hydrate (NaH2PO4 H2O), sodium hydrogen phosphate (Na2HPO4), 6-mercapto-1-hexanol (C6–OH), and tris(2-carboxyethyl) phosphine hydrochloride (TCEP), were purchased from Sigma-Aldrich (St. Louis, MO, USA). The anti-miRNA-29a DNA probe (5'-TAACCGATTTCAGATGGTGCTA-3'), labeled with methylene blue (MB) at 3', as the redox reporter, was purchased from Biosearch Technologies (Novato, CA, USA). The miRNA-29 target was purchased from IDT - Integrated DNA Technologies (Coralville, IA, USA). The DNA target (riportare la sequenza) and other sequences tested as interferents, were purchased from Biosearch Technologies (Novato, CA, USA). All the electrochemical measurements were carried out using a portable potentiostat PalmSens 4 (PalmSens, Netherlands) equipped with a multi-8 reader and connected to a laptop.

Screen-printed platform

Electrodes were in-house produced onto a flexible polyester film as substrate (HT5, Autostat). The adoption of polyester-based substrates for manufacturing electrochemical strips compared to paper-based ones allow creating more robust platforms to be applied in decentralized context. The three-electrode design was manually screen printed using a squeegee to spread the conductive inks through an ad-hoc designed mask. Briefly, Ag/AgCl ink (Electrodag 477 SS, Acheson, Italy) was used to print the connections and the reference electrode,

and the carbon ink (Electrodag 421, Acheson, Italy) was used to print the working and counter electrodes. After the inks were printed, the strips were thermally cured, 80 °C for 15 min, is necessary to make the printed ink stable for electrochemical measurements. The diameter of the working electrode was 0.4 cm, and the electrochemical strips were ca. 2.5 cm (height) x 1 cm (width). The possible diffusion of aqueous samples to connector was avoided by placing an adhesive tape to define the testing area.

Microscale thermophoresis (MST) experiments

The concentration of the labeled oligonucleotide was kept constant at 200 nM, while a serial dilution of the investigated DNA/RNA targets was prepared and mixed with the labeled oligonucleotide solution with a volume ratio of 1:1. Each of the 16 solutions of a titration was filled into a capillary, which were measured successively to create the respective data points in the experiment. General settings were applied for all measurements as follows: LED laser: red; fluorescence measurement before MST: 3 s; MST (IR laser) on: 21 s; fluorescence after MST: 1 s. Measurements were performed at 25 °C, using 100% LED and medium MST power to achieve an appropriate thermophoretic response. MST measurements were analyzed using the MO. Affinity Analysis software (v2.3) provided with the instrument. For each titration experiment, normalized fluorescence ratios (hot/cold x 1000, FNorm) were plotted versus the respective concentration (expressed in log scale) to obtain the binding curves. Plots were rendered using Origin 7.0 software (OriginLab Corp., Northampton, MA, USA).

AuNPs synthesis

First of all, glassware and magnetic stir bar used in this synthesis were cleaned in aqua regia (HCl/HNO_3 3:1 (v/v)), rinsed in distilled water, and subsequently cleaned with piranha solution (H_2SO_4/H_2O_2 7:3 (v/v)), and rinsed again with distilled water before use. Then, AuNPs were obtained in a round flask at room temperature (RT), mixing 9 mL of distilled water with 1 mL of 0.01 g/mL $HAuCl_4$ and 2 mL of 0.01 g/mL sodium citrate. Then, 0.5 mL of 20 mM sodium borohydride were added drop-by-drop. The solution was left under stirring and in dark condition overnight. The AuNPs dispersion was then stored at 4 °C.

AuNPs characterization

The synthesized AuNPs have been characterized through scanning electron microscopy, dynamic light scattering and energy-dispersive X-ray spectroscopy were carried out attaching the experiments. DLS measurements revealed a monodispersed AuNPs suspension as reported in Figure S1 with an average diameter of 196.2± 20.65 nm and a PDI of 0.13± 0.07, highlighting the synthesis of uniform and monodisperse particles

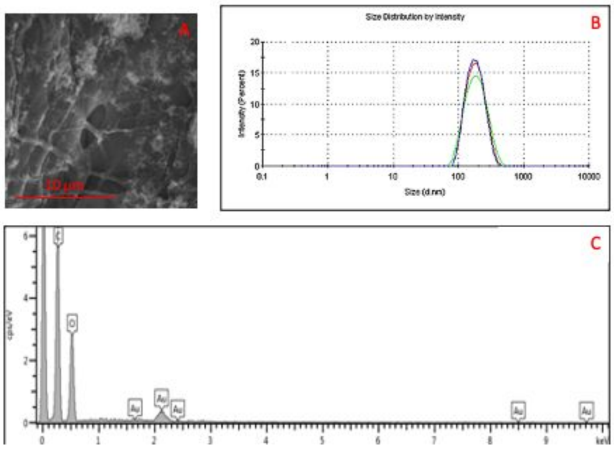

Figure S1. A) SEM images of the modified SPE, B) AuNPs size distribution (n = 3), C) EDS analysis of the SPE modified with AuNPs.

Experimental procedure

The concentration of supporting electrolyte has been finely optimized choosing among various level of sodium chloride, e.g. 150, 250, 500 and 1000 mM, and 1000 mM of NaCl was consistent with the highest sensitivity of the device. In addition, the presence of MgCl2 helped the formation of duplex structure, furtherly improving the performance. The presence of complimentary target was capable of forming a duplex structure with the immobilized probe, thus leading to a decrease of the electrochemical signal (signal off). The signal was recorded after 30 minutes using Square Wave Voltammetry (SWV) as electrochemical technique (equilibration time = 5 s, E begin = 0.0 V, E end = −0.5 V, E step = 0.001 V, Amplitude = 0.01 V, Frequency = 50.0 Hz).

Matrix effect of measurement in serum

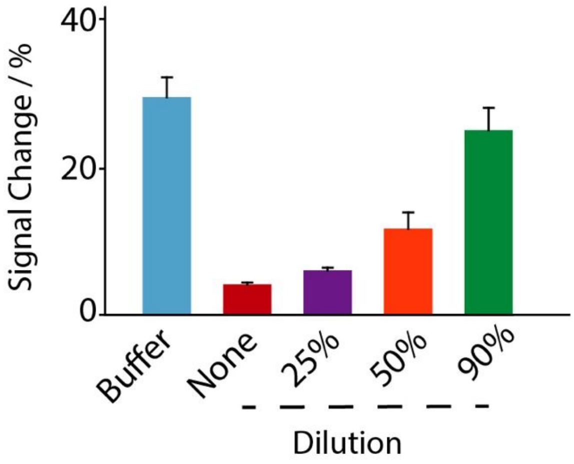

Figure S2. Matrix effect in presence of 10 nM miRNA-29a analyzed with the optimized platform in buffer, whole serum, 25%-diluted serum, 50%-diluted serum and 90%-serum. All the bars have been obtained as the mean of three replicates.

Sequences used for selectivity studies

5’-AAACUUUUGGGGAUGACGA-3’

5’-ACACUUCCGCGCGAUGACGCC-3’

5’-TATCCCATTTAGACTACTA-3’

5’-TATCCATTAGACTACTACGCA-3’
